# Double Burden of Distress: Exploring the Joint Associations of Loneliness and Financial Strain with Suicidal Ideation During the COVID-19 Pandemic in Canada

**DOI:** 10.3390/ijerph22050682

**Published:** 2025-04-25

**Authors:** Fahima Hassan, Lihui Liu, Cindy Feng

**Affiliations:** Department of Community Health and Epidemiology, Faculty of Medicine, Dalhousie University, Halifax, NS B3H1V7, Canada; fahima.hassan@dal.ca (F.H.); lihui.liu@dal.ca (L.L.)

**Keywords:** mental health, suicidal ideation, COVID-19 pandemic, loneliness, financial strain

## Abstract

Background: The COVID-19 pandemic, coupled with social distancing measures and economic disruptions, has been associated with increased experiences of loneliness and financial strain. While prior research has examined their separate associations with suicidal ideation, limited attention has been given to their joint relationship. Methods: We used data from the 2022 Mental Health and Access to Care Survey (MHACS) (n = 9861; ages 15+ in Canada) to assess whether financial strain modifies the association between loneliness or emotional distress and suicidal ideation. Multivariable survey-weighted logistic regression was conducted, adjusting for sociodemographic, economic, psychosocial, and health-related characteristics, including mental health and substance use conditions. Results: Among the 9743 respondents who answered the question on suicidal ideation, 355 (3.65%) reported suicidal ideation. Compared to individuals with neither stressor, those who experienced loneliness or emotional distress alone had 1.54 times higher odds of suicidal ideation (aOR = 1.54, 95% CI: 1.29–1.84, *p* < 0.001), while those who reported financial strain alone had 0.58 times the odds (aOR = 0.58, 95% CI: 0.43–0.80, *p* = 0.001). The highest odds were observed among individuals who experienced both loneliness/emotional distress and financial strain, with an adjusted odds ratio of 2.05 (95% CI: 1.71–2.45, *p* < 0.001), indicating an interaction between these stressors. Conclusion: The co-occurrence of loneliness or emotional distress and financial strain was associated with higher odds of suicidal ideation during the COVID-19 pandemic, compared to individuals experiencing neither stressor. These findings highlight the importance of considering both social and economic stressors when assessing mental health risks. Given the cross-sectional nature of this study, further longitudinal research is needed to explore the temporal relationships and potential causal pathways linking these experiences to suicidal ideation.

## 1. Introduction

The COVID-19 pandemic profoundly disrupted social and economic structures worldwide, significantly amplifying risks to mental health. Public health measures, including social distancing, aimed at limiting SARS-CoV-2 spread, exacerbated loneliness, isolation, and emotional distress [1,2,3]. Simultaneously, economic challenges such as recessions, unemployment, and financial instability exacerbated financial strain and insecurity [4]. Both loneliness and financial stress are well documented as key risk factors for suicidal ideation, a critical indicator of severe mental distress and a precursor to suicide [4,5,6,7].

In Canada, these mental health challenges were similarly pronounced. According to data from the Survey on COVID-19 and Mental Health, the prevalence of suicidal ideation rose from 2.7% in 2019 to 3.8% in 2023 [8]. National public health surveillance reports suggest that pandemic-related social and economic stressors were significantly associated with this increase [9]. However, limited research has examined how these stressors may co-occur and relate to suicidal ideation in the Canadian context. Given Canada’s unique social and health policy landscape during the pandemic, it is important to explore how the combined presence of emotional and financial stressors may be associated with population mental health outcomes.

Loneliness, a central component of emotional distress, entails a range of negative emotions, including grief, anxiety, despair, worry, and stress [10]. Even before the COVID-19 pandemic, loneliness had been recognized as a significant psychosocial risk factor for suicidal ideation and behaviour [11,12,13]. The pandemic exacerbated these experiences, leading to a notable increase in loneliness across the population, which further amplified the risk of suicidal ideation [3,4,14,15,16,17]. Research indicates that the emotional strain induced by isolation during the pandemic, particularly among individuals who experienced social disconnection, significantly contributed to the heightened risk of suicidal thoughts. For instance, studies conducted by the Public Health Agency of Canada (PHAC) revealed that individuals reporting loneliness or isolation during the pandemic were nearly eight times as likely to experience suicidal ideation compared to those who did not [9]. This is further supported by national data showing that feelings of loneliness were independently associated with increased odds of suicidal ideation during the pandemic [18].

Similarly, financial strain has long been recognized as a significant contributor to suicidal ideation [19,20,21,22,23], a situation exacerbated during the pandemic by economic disruptions such as job losses, reduced income, and financial insecurity [1,4,6]. According to PHAC, individuals who lost their jobs or income during the pandemic were twice as likely to experience suicidal ideation compared to those who did not [9]. The same national survey also found that individuals facing pandemic-related income- or job loss had a significantly higher likelihood of suicidal thoughts than those who were not economically affected [18]. Together, these findings underscore the critical role of both social and financial stressors in exacerbating mental health challenges during times of societal crisis.

While loneliness and financial stress have each been independently associated with increased suicidal ideation, their combined association during a global crisis such as the COVID-19 pandemic remains insufficiently explored. The simultaneous magnification of these stressors likely created a “double burden”, particularly for individuals already vulnerable due to pre-existing mental health conditions, socioeconomic disadvantages, or marginalized statuses. Stress processes and cumulative risk frameworks suggest that co-occurring adversities may not simply add to one another but can interact in a multiplicative way, compounding psychological harm. In this context, financial strain may amplify the emotional toll of loneliness by limiting access to vital coping resources, such as mental health support, social networks, and basic necessities [6,24]. At the same time, emotional distress can impair functioning, reducing job performance and increasing the likelihood of employment disruptions, which, in turn, further undermine financial stability [25,26,27]. This cyclical interplay between emotional and financial stressors heightens vulnerability to suicidal ideation.

This study hypothesizes that the interaction between emotional distress and financial strain is associated with an increased likelihood of suicidal ideation during the COVID-19 pandemic. To address this gap, it examines how the co-occurrence of these stressors is related to suicidal ideation in the Canadian population. By adjusting for key confounders—including sociodemographic and economic characteristics, as well as pre-existing health conditions—the study aims to offer insight into the cumulative burden of these stressors and inform strategies to better support populations at heightened risk.

## 2. Materials and Methods

### 2.1. Study Design and Data Source

This study utilized data from the Mental Health and Access to Care Survey (MHACS), a cross-sectional survey conducted by Statistics Canada [28]. The survey included respondents aged 15 years and older, surveyed between 17 March and 31 July 2022, across all ten Canadian provinces and territories. Individuals living on Aboriginal reserves, in certain remote areas, full-time members of the Canadian Armed Forces, and institutionalized populations were excluded from the study.

The MHACS was designed to assess changes in mental health status and access to mental health services before and during the COVID-19 pandemic. It provides a comprehensive dataset on various mental health indicators, including emotional distress, mental health service use, and associated demographic and socio-economic factors.

The initial sample consisted of 39,485 households, identified using the 2021 Census as a sampling frame. A subset of these households was selected for the long questionnaire, resulting in 9861 respondents. The population was stratified to facilitate the oversampling of visible minority groups (South Asian, Black, Chinese, Filipino) and to ensure sufficient representation within specified age and gender groups. The purpose of this stratification was to collect data from enough individuals to allow for meaningful analyses at the subgroup level. After applying two stages of selection (household and one individual respondent per household), the final sample size was 9861, with an overall response rate of 25%. Sampling weights were calculated by Statistics Canada to adjust for design effects (e.g., stratification) and non-response. According to Statistics Canada, “It is expected that the population was well-covered, due to the high response rate to the 2021 Census”.

The design, methodology, and sampling frame of the MHACS have been detailed in previous official reports published by Statistics Canada [28].

### 2.2. Study Variables and Measures

#### 2.2.1. Outcome Variable

The outcome variable, suicidal ideation, was a binary variable based on self-reported data. Respondents were categorized as having experienced serious suicidal thoughts in the past 12 months or not, regardless of any prior history of suicidal ideation. Although the full MHACS dataset includes additional variables related to suicide plans and suicide attempts, these were not available in the Public Use Microdata File (PUMF) released by Statistics Canada. Therefore, our analysis focused on past-year suicidal ideation as the sole outcome indicator.

#### 2.2.2. Exposure Variables

The MHACS included a set of items assessing various pandemic-related stressors, each scored using a binary (Yes/No) format. For this study, we focused on two specific stressors:

− Loneliness or emotional distress: Capturing experiences of loneliness, isolation, or emotional distress (e.g., grief, anger, worry).
− Financial strain: Reflecting difficulty in meeting financial obligations or essential needs (e.g., rent, mortgage, utilities, groceries).

#### 2.2.3. Covariates

A range of covariates were included in the analysis to account for factors that may confound the relationship between pandemic-related stressors and suicidal ideation. These covariates were categorized as follows:

##### Sociodemographic and Economic Factors

- Gender: Coded as men+ and women+. Non-binary respondents were reclassified into these categories using a random imputation method provided by MHACS.
- LGBTQ2+ status: LGBTQ2+ vs. non-LGBTQ2+.
- Age: Categorized into the following groups: 15–19, 20–24, 25–29, 30–34, 35–44, 45–54, 55–64, and 65 years or older.
- Marital status: Classified as married/living common law; single (i.e., never married or lived common law); or divorced/widowed.
- Place of residence: Based on population size—rural area (<1000), small population centre (1000–29,999), medium population centre (30,000–99,999), and large urban centre (≥100,000).
- Education level: Less than high school diploma or equivalent, high school diploma or equivalent, trades/college/university certificate or diploma below the bachelor’s level, or bachelor’s degree and above.
- Household income: Low (<CAD 30,000), middle (CAD 30,000–CAD 99,999), and high (≥CAD 100,000).
- Prescription medication insurance: Whether the respondent had insurance coverage for prescription medications (Yes/No).

##### Mental and Substance Use Disorders

Mental and substance use disorders were assessed using the Canadian adaptation of the World Health Organization Composite International Diagnostic Interview (WHO-CIDI) [29], a standardized, structured diagnostic tool that has been widely validated for epidemiological research. The WHO-CIDI is designed to assess psychiatric conditions based on diagnostic criteria from the Diagnostic and Statistical Manual of Mental Disorders (DSM) and International Classification of Diseases (ICD), offering a consistent method for identifying mental health and substance use disorders in large population-based surveys. Importantly, while clinical diagnoses are typically made by healthcare professionals during face-to-face consultations, the WHO-CIDI uses a structured interview format administered by trained interviewers to identify whether an individual meets the diagnostic criteria for a particular mental health condition. This approach provides a reliable method for diagnosing a range of disorders in a non-clinical, survey setting.

For the purpose of this study, we focused on past-year diagnoses (i.e., disorders that were present at any point in the 12 months prior to the survey), as this timeframe aligns with the pandemic period, ensuring the relevance of the findings to the research question. This differs from lifetime diagnoses, which reflect any occurrence of a condition during the respondent’s entire life.

The disorders assessed included:

- Mood disorders (e.g., Major Depressive Episode, Bipolar I, Bipolar II, Hypomania).
- Anxiety disorders (e.g., Generalized Anxiety Disorder, Social Anxiety Disorder).
- Substance use disorders (e.g., alcohol, cannabis, and other drug use disorders).

Each condition was categorized as a binary variable (Yes/No), indicating whether or not the respondent met the diagnostic criteria for that disorder in the past 12 months.

##### Psychosocial Factors

- Social belonging: Measured by respondents’ self-reported sense of belonging to their local community. Response options included very strong, somewhat strong, somewhat weak, or very weak.

##### Physical and Functional Health Factors

- Chronic physical or mental health conditions: Presence of long-term health conditions diagnosed by a healthcare professional, including schizophrenia, psychosis, eating disorders, or other chronic physical or mental conditions (Yes/No).
- Functional impairment: Assessed using the WHO Disability Assessment Schedule (WHODAS) 2.0, which evaluates limitations across six domains, including mobility, self-care, and social participation. Scores range from 0 to 40, with higher scores indicating greater functional impairment.

### 2.3. Statistical Analysis

We conducted survey-weighted descriptive statistics to examine the distribution of key variables, including COVID-19-related emotional distress and financial strain, stratified by the presence or absence of suicidal ideation in the past 12 months. These analyses accounted for the complex survey design and provided an overview of how these factors were distributed among respondents in relation to suicidal ideation. Categorical variables were summarized as frequencies and percentages, while continuous variables were summarized using mean and standard deviation (SD). Comparisons were made between respondents with and without suicidal ideation to assess the association between these variables.

Prior to the multivariable analysis, we performed bivariate weighted logistic regression to explore unadjusted associations between suicidal ideation and the key exposure variables—COVID-19-related loneliness or emotional distress and financial strain—as well as relevant covariates. We also tested for statistical interaction between the two exposures to examine whether their co-occurrence was associated with different odds of suicidal ideation compared to each exposure independently. Subsequently, we conducted a multivariable survey-weighted logistic regression to estimate adjusted associations with suicidal ideation, accounting for all relevant covariates.

We assessed the assumptions underlying the multivariable analysis. For continuous covariates (i.e., functional impairment score), we examined the linearity assumption using diagnostic plots and confirmed that the logit of the outcome was approximately linear. Multicollinearity was assessed using variance inflation factors (VIFs), with all variables showing VIFs well below 10, indicating no evidence of problematic multicollinearity. Although some variables had missing data, the overall proportion of missingness was low (not exceeding 8% for any variable). We did not implement formal imputation methods; however, given the low level of missingness and the use of survey weights designed to account for non-response at the design level, any resulting bias is likely to be minimal.

All analyses were conducted using Stata version 18 (StataCorp LLC, College Station, TX, USA). To account for the complex sampling design of the MHACS, we applied both the survey weights and 1000 bootstrap weights provided by Statistics Canada. The survey weights accounted for unequal probabilities of selection and non-response, ensuring representativeness of the Canadian population. The bootstrap weights were used to obtain more reliable standard errors and 95% confidence intervals, reflecting the survey’s stratified and clustered design.

## 3. Results

Table 1 presents the weighted descriptive characteristics of the study population by suicidal ideation status. Overall, 3.65% of respondents reported suicidal ideation. A greater proportion of individuals who reported COVID-19-related loneliness or emotional distress (77.80%) and financial strain (39.79%) also reported suicidal thoughts, compared to those who did not. Higher proportions of suicidal ideation were observed among younger age groups, individuals identifying as LGBTQ2+, those who were single, and those with lower levels of education or income. Respondents with mood or anxiety disorders, substance use disorders, lower levels of social belonging, or chronic physical or mental health conditions also made up relatively higher percentages of those reporting suicidal ideation.

The univariate analysis (Table 2) revealed significant crude associations between COVID-19 pandemic-related stressors and suicidal ideation. Loneliness or emotional distress was notably associated with higher odds of suicidal ideation, with individuals reporting these experiences having 3.73 times the odds compared to those without (OR: 3.73, 95% CI: 3.32–4.19, *p* < 0.001). Financial strain, such as job or income loss, was also associated with greater odds of suicidal ideation (OR: 2.16, 95% CI: 1.97–2.37, *p* < 0.001). These results indicate that, when examined individually, pandemic-related stressors were strongly associated with suicidal ideation.

Multivariable survey-weighted logistic regression was conducted, adjusting for social, demographic, economic, psychosocial, and health-related factors, as well as mental health and substance use conditions (Table 3). Compared to individuals with neither stressor, those experiencing loneliness or emotional distress in the absence of financial strain had 1.54 times higher odds of suicidal ideation (aOR = 1.54, 95% CI: 1.29–1.84, *p* < 0.001), while those experiencing financial strain alone had lower odds (aOR = 0.58, 95% CI: 0.43–0.80, *p* = 0.001). The odds were highest among those experiencing both loneliness or emotional distress and financial strain, with an adjusted odds ratio of 2.05 (95% CI: 1.71–2.45, *p* < 0.001). These findings suggest a synergistic association between loneliness/emotional distress and financial strain, with their co-occurrence being associated with substantially higher odds of suicidal ideation compared to each factor alone.

Demographic and socioeconomic characteristics were significantly associated with suicidal ideation. LGBTQ2+ individuals had notably higher odds of reporting suicidal ideation. Younger age groups, particularly those aged 15 to 29, exhibited the highest odds, with decreasing odds observed in older age groups. Individuals who were single, divorced, or widowed had higher odds compared to those who were married or in common-law relationships. Respondents living in large urban areas had lower odds of suicidal ideation compared to those in smaller or rural areas. Higher education levels and having insurance coverage for prescription medications were associated with lower odds of suicidal ideation.

Mental health conditions showed strong associations with suicidal ideation. Individuals with mood disorders (aOR = 4.41, 95% CI: 3.79–5.13), anxiety disorders (aOR = 1.83, 95% CI: 1.57–2.13), and substance use disorders (aOR = 1.50, 95% CI: 1.26–1.79) had significantly higher odds of reporting suicidal ideation. Psychosocial factors, such as social belonging, were also important; respondents who reported “very weak” social belonging had more than twice the odds of suicidal ideation compared to those with stronger social connections (aOR = 2.81, 95% CI: 2.18–3.63). Additionally, long-term physical or mental health conditions and higher WHO disability scores were associated with increased odds, highlighting the importance of addressing both mental and physical health challenges in suicide prevention strategies.

In addition to the odds ratios, we further examined the conditional prevalence estimates for suicidal ideation across the four combinations of loneliness or emotional distress and financial strain (see Table 4). The results suggest notable variations in the likelihood of suicidal ideation based on the presence or absence of each stressor.

- Neither stressor (No emotional distress, no financial strain): The estimated prevalence of suicidal ideation in this group was 2.41% (95% CI: 2.12%–2.70%).
- Emotional distress or loneliness alone (No financial strain, yes emotional distress): The estimated prevalence increased to 3.47% (95% CI: 3.19%–3.76%).
- Financial strain alone (Yes financial strain, no emotional distress): The estimated prevalence was 1.51% (95% CI: 1.12%–1.89%).
- Both stressors (Yes financial strain, yes emotional distress): The estimated prevalence of suicidal ideation in this group was substantially higher at 4.37% (95% CI: 3.98%–4.76%).

**Table 4:** Conditional prevalence of suicidal ideation by combinations of loneliness/emotional distress and financial strain.

Combination of Stressors	Prevalence of Suicidal Ideation	95% CI
Neither Stressor (No emotional distress, No financial strain)	2.41%	2.12%–2.70%
Emotional Distress Only (No financial strain, Yes emotional distress)	3.47%	3.19%–3.76%
Financial Strain Only (Yes financial strain, No emotional distress)	1.51%	1.12%–1.89%
Both Stressors (Yes financial strain, Yes emotional distress)	4.37%	3.98%–4.76%

These conditional prevalence estimates highlight that the combined presence of both emotional distress and financial strain was associated with a markedly higher likelihood of suicidal ideation than the presence of either factor alone. Specifically, the presence of both stressors nearly doubled the prevalence of suicidal ideation compared to when only one of the stressors was present.

**Figure 1:**
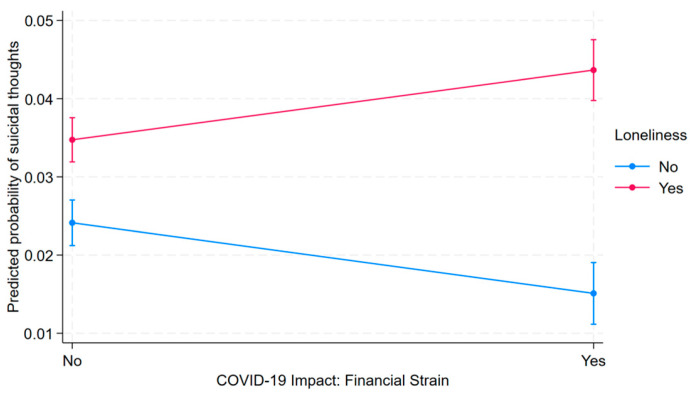
Predicted probability of suicidal ideation by COVID-19-related financial strain and loneliness/emotional distress.

To examine the joint association of COVID-related financial hardship and loneliness/emotional distress with suicidal ideation, we conducted pairwise comparisons of predicted probabilities of suicidal ideation using Bonferroni-adjusted significance tests (Table 5). Individuals reporting both financial hardship and loneliness had the highest predicted prevalence—approximately 2.86 percentage points higher than those reporting financial hardship alone (*p* < 0.001). Although the magnitude of this difference may appear modest, it was statistically significant, indicating a robust association. Given the serious nature of the outcome, even small increases in predicted prevalence may be meaningful, particularly at the population level. Compared to those reporting neither stressor, the predicted prevalence was 1.95 percentage points higher among individuals with both stressors (*p* < 0.001), 1.06 points higher among those with loneliness alone (*p* < 0.001), and 0.90 points lower among those with financial hardship alone (*p* = 0.001). These findings underscore the heightened prevalence of suicidal ideation associated with the co-occurrence of financial and emotional stressors. Figure 1 visually presents the predicted probabilities across the four stressor combinations, highlighting the elevated prevalence linked to their joint presence.

## 4. Discussion

This study explored the association between suicidal ideation and two major COVID-19-related stressors: loneliness or emotional distress and financial strain. Loneliness or emotional distress, in the absence of financial strain, was associated with a 1.54-fold increase in the odds of suicidal ideation (aOR = 1.54, 95% CI: 1.29–1.84, *p* < 0.001). In contrast, financial strain alone was associated with lower odds of suicidal ideation (aOR = 0.58, 95% CI: 0.43–0.80, *p* = 0.001), suggesting that its isolated association with suicidal ideation might not be as strong. However, when both emotional distress and financial strain were present, the odds of suicidal ideation were significantly higher, more than doubling the odds (aOR = 2.05, 95% CI: 1.71–2.45, *p* < 0.001). This suggests an interaction between emotional distress and financial strain, where the co-occurrence of both stressors was associated with a substantially higher likelihood of suicidal ideation than either factor alone.

The predicted prevalence of suicidal ideation across the four combinations of emotional distress and financial strain offers further insight into their joint association with suicidal thoughts. The highest prevalence was observed among individuals experiencing both stressors (4.37%), underscoring the cumulative burden of co-occurring emotional and financial difficulties. Notably, those reporting financial strain in the absence of emotional distress had a relatively low prevalence (1.51%), suggesting that financial strain alone may not be strongly associated with suicidal ideation. In contrast, emotional distress alone was associated with a higher prevalence (3.47%), and this association was further amplified when combined with financial strain. Although the overall prevalence of suicidal ideation was relatively low across all groups, pairwise comparisons revealed statistically significant differences between all four groups. This indicates that, despite the modest absolute prevalence rates, the differences in suicidal ideation between groups are meaningful and unlikely to be due to random variation. These results underscore the importance of accounting for the co-occurrence of emotional distress and financial strain when assessing suicide risk.

These findings are consistent with a growing body of evidence suggesting that both financial strain and emotional distress are related to an increased likelihood of suicidal ideation. Financial difficulties—such as struggling to meet basic needs or maintain financial stability—are linked to feelings of hopelessness, helplessness, and loss of control, all of which are associated with suicidal thoughts [1,5,8,14,30,31,32]. Additionally, financial strain can limit access to essential coping resources, including mental health services and social support, which may heighten vulnerability to emotional distress [6,24].

On the other hand, loneliness and emotional distress can exacerbate the perception of financial hardship, creating a cycle of psychological and economic adversity. Individuals experiencing emotional distress may perceive their financial situation as more overwhelming, which exacerbates both emotional suffering and material insecurity. Mental health conditions have also been linked to lower employment rates, further compromising financial stability and restricting access to critical resources [25,26,27].

Financial strain and emotional distress often co-occur and may be linked to a broader constellation of challenges that contribute to elevated psychological risk. For example, tangible consequences of financial hardship, such as difficulties affording food or stable housing, can deepen social isolation and feelings of despair. These stressors can, in turn, impair cognitive functioning, reduce problem-solving abilities, and lower motivation to seek help, particularly for those already dealing with emotional distress or loneliness [33]. While our study design does not allow us to determine causality or directionality, the observed associations suggest a complex, potentially compounding relationship between financial and emotional stressors. Notably, the combination of both was associated with a greater risk of suicidal ideation than either factor alone [31].

Although causality cannot be inferred from our cross-sectional data, the observed associations occurred during the COVID-19 pandemic—a period marked by widespread social and economic disruption. Public health responses such as lockdowns, social distancing mandates, and emergency financial supports may have shaped individuals’ exposure to or experience of emotional and financial stress. These findings underscore the importance of integrating both economic and mental health considerations into public health planning. Interventions that address the co-occurrence of financial hardship and emotional distress may be particularly effective in mitigating mental health risks during future public health emergencies.

In addition to COVID-19-related stressors, our study identified several other significant factors associated with suicidal ideation. Notably, LGBTQ2+ individuals were found to be at substantially higher risk, aligning with prior research that highlights the role of stigma, discrimination, and social marginalization in increasing suicide risk within this population [17,34,35]. These findings emphasize the urgent need for targeted mental health support for marginalized groups, particularly during times of crisis. Younger individuals were also at greater risk for suicidal ideation, as were those who were divorced or widowed compared to those who were married. This suggests that developmental factors, disruptions in social relationships, and life transitions contribute significantly to the vulnerability of these groups. Interventions tailored specifically to younger populations, particularly within educational settings, could be instrumental in mitigating suicide risk during pandemics or similar crises [36,37,38]. Finally, individuals with mood, anxiety, or substance use disorders and chronic physical or mental health conditions were at an increased odds of suicidal ideation, consistent with the established literature [39,40]. These findings reinforce the critical need for comprehensive mental health interventions, particularly for those with pre-existing mental health conditions, to effectively reduce the risk of suicidal ideation during crises.

### Strengths and Limitations

This study has several notable strengths. First, the study draws on a large, nationally representative dataset collected across Canada, enhancing the generalizability of our findings to the wider Canadian population. Second, the focus on COVID-19-related stressors provides timely and policy-relevant insights into how the pandemic may have influenced mental health outcomes, particularly suicidal ideation, under real-world public health and economic conditions. Third, by considering both stressors simultaneously, this research highlights the critical interplay between emotional and economic factors, which have been underexplored in prior studies. Fourth, we adjusted for a wide range of potential confounders, including mental health variables and demographic, social, and economic factors, which adds precision to our estimates.

However, this study has several important limitations. First, vulnerable populations, such as those facing technological barriers (e.g., limited internet access) and individuals experiencing homelessness, may have been underrepresented due to the survey-based data collection method. Additionally, the sample excluded individuals from territories, reserves, and institutional settings, potentially leading to the underrepresentation of key subgroups, including Indigenous communities. These sampling biases limit the generalizability of our findings to the broader population. Furthermore, other unmeasured and unadjusted factors could have impacted the precision of our results.

Second, while the proportion of missing data across variables was relatively low (not exceeding 8%), we did not apply formal imputation methods. Instead, we relied on survey-weighted analyses to partially address potential biases associated with missingness. Nonetheless, if the data were not missing at random, our findings may still be subject to bias. Future studies could benefit from employing multiple imputation techniques or conducting sensitivity analyses to assess the robustness of results under different missing data assumptions.

Third, the study relied on single-time, binary indicators for both exposure and outcome variables. COVID-related stressors, such as financial strain and loneliness, were measured using single-item or binary variables, which may not capture the full complexity or multidimensional nature of these constructs. In future research, the use of validated instruments—such as the Psychological Inventory of Financial Scarcity for financial stress [41] and the UCLA Loneliness Scale for loneliness [42]—could be helpful. Moreover, the use of instruments specifically designed to measure suicidal ideation, like the Beck Scale for Suicide Ideation [43] or Columbia-Suicide Severity Rating Scale [44], could provide more reliable and detailed outcomes.

Moreover, while emotional distress and loneliness were highly prevalent among participants reporting suicidal ideation (present in approximately 77.8% of such cases), it is important to distinguish between these overlapping but conceptually distinct constructs. Emotional distress and loneliness reflect broader affective and psychosocial vulnerabilities that may predispose individuals to suicidal thoughts, but they are not synonymous with suicidal ideation. The high co-occurrence may reflect shared underlying risk factors or the temporal proximity of distress symptoms to suicidal thinking. This overlap limits our ability to draw firm conclusions about divergent validity within a cross-sectional design. We recommend that future research adopts longitudinal frameworks and uses validated, multi-item measures to better disentangle these constructs.

Finally, while we adjusted for a comprehensive set of potential confounders to reduce bias from confounding factors, it is important to consider that adjusting for multiple covariates may have attenuated some associations, particularly when certain variables act as mediators in the pathway to suicidal ideation. These adjustments could have influenced the strength of the observed relationships. The cross-sectional design of this study further limits the ability to draw causal conclusions. Although significant associations were observed between loneliness/emotional distress, financial strain, and suicidal ideation, the directionality of these relationships remains unclear. Longitudinal studies are needed to clarify potential causal pathways—specifically, to examine whether financial strain acts as an antecedent to emotional distress, rather than a moderator, or whether loneliness and emotional distress serve as mediators in the pathway from financial hardship to suicidal ideation. It is also plausible that emotional distress and social isolation contribute to financial difficulties by impairing occupational functioning or reducing access to supportive networks, thereby indirectly increasing the risk of suicidal ideation. Future studies with longitudinal designs would be valuable for exploring these complex dynamics more deeply and refining our understanding of these potentially bidirectional interactions.

Despite these limitations, our study provides important insights into the co-occurrence of emotional and financial stressors and their associations with suicidal ideation during the COVID-19 pandemic. These findings highlight the importance of considering both emotional and economic challenges when examining patterns of suicidal ideation, particularly during periods of widespread societal disruption. Further research using longitudinal designs is warranted to better understand how these factors may interact over time and contribute to informing future prevention strategies.

## 5. Conclusions

This study examined the association between emotional distress, financial strain, and suicidal ideation among Canadian adults during the COVID-19 pandemic. The findings indicate that individuals reporting both emotional distress and financial strain had a higher prevalence and odds of suicidal ideation than those reporting either stressor alone. While causality cannot be inferred due to the cross-sectional design, these results underscore the importance of considering the co-occurrence of psychosocial and economic stressors in suicide risk assessments. Future research using longitudinal data is needed to better understand the directionality and mechanisms underlying these associations and to inform evidence-based strategies for mental health support during public health crises.

## Figures and Tables

**Table 1:** Weighted sample characteristics of study respondents stratified by suicidal ideation in the past 12 months, MHACS.

Variables	Suicidal Ideation				
	Total (%) 9743 (100%)	Yes (%) 355 (3.65%)		No (%) 9387 (96.35%)	
COVID-19 pandemic stressors					
Loneliness or emotional distress (n = 9693)					
Yes	4800 (49.52%)	272	77.80%	4528	48.46%
No	4893 (50.48%)	78	22.20%	4815	51.54%
Financial strain (n = 9693)					
Yes	2328 (24.01%)	139	39.79%	2189	23.42%
No	7365 (75.99%)	211	60.21%	7155	76.58%
Social demographic and economic factors					
Gender (n = 9728)					
Men+	4751 (48.84%)	154	43.56%	4597	49.04%
Women+	4977 (51.16%)	200	56.44%	4777	50.96%
LGBTQ2+ status (n = 9492)					
LGBTQ2+	616 (6.49%)	94	27.33%	522	5.71%
Non-LGBTQ2+	8876 (93.51%)	249	72.67%	8627	94.29%
Age (n = 9743)					
15 to 19 years	654 (6.71%)	59	16.51%	595	6.34%
20 to 24 years	720 (7.39%)	59	16.52%	661	7.04%
25 to 29 years	688 (7.06%)	40	11.35%	648	6.90%
30 to 34 years	792 (8.13%)	42	11.74%	751	8.00%
35 to 44 years	1698 (17.43%)	53	14.81%	1646	17.53%
45 to 54 years	1316 (13.51%)	38	10.59%	1279	13.62%
55 to 64 years	1721 (17.67%)	41	1.16%	1680	17.89%
65 years or older	2153 (22.09%)	24	6.89%	2128	22.67%
Marital status (n = 9688)					
Married or living common law	5541 (57.19%)	111	3.18%	5430	58.14%
Single	2842 (29.34%)	188	53.93%	2654	28.42%
Divorced or widowed	1305 (13.47%)	50	14.27%	1256	13.45%
Rurality (n = 9743)					
Rural area (less than 1000)	1760 (18.06%)	59	16.49%	1701	18.12%
Small population centre (1000 to 29,999)	1086 (11.15%)	36	10.12%	1050	11.19%
Medium population centre (30,000 to 99,999)	869 (8.92%)	44	12.44%	825	8.79%
Large urban population centre (≥100,000)	6027 (61.87%)	217	60.95%	5811	61.90%
Education (n = 9551)					
Less than high school diploma/certificate	1024 (10.72%)	69	20.18%	954	10.37%
Highschool diploma/certificate	2362 (24.73%)	112	32.66%	2250	24.44%
Trade/college/university diploma/certificate	3231 (33.83%)	83	24.29%	3148	34.19%
Bachelor’s degree or higher	2934 (30.72%)	78	22.86%	2856	31.01%
Household income (n = 9669)					
Low income (<CAD 30,000)	662 (6.85%)	29	8.15%	633	6.80%
Middle income (CAD 30,000 to CAD 99,999)	3954 (40.89%)	156	44.05%	3797	40.77%
High income (≥CAD 100,000)	5054 (52.26%)	169	47.80%	4884	52.43%
Insurance coverage for prescription medicine (n = 9559)					
Yes	7158 (74.88%)	246	72.19%	6912	74.98%
No	2401 (25.12%)	95	27.81%	2306	25.02%
Mental health and substance use disorders					
Mood disorders (n = 9411)					
Yes	785 (8.34%)	175	52.04%	610	6.73%
No	8626 (91.66%)	161	47.96%	8465	93.27%
Anxiety disorders (n = 9272)					
Yes	937 (10.11%)	153	44.85%	784	8.78%
No	8335 (89.89%)	188	55.15%	8147	91.22%
Substance use disorders (n = 9019)					
Yes	341 (3.78%)	50	15.44%	291	3.34%
No	8678 (96.22%)	272	84.56%	8406	96.66%
Psychosocial factors					
Social belongings (n = 9440)					
Very strong	1457 (15.44%)	24	7.25%	1433	15.74%
Somewhat strong	4528 (47.97%)	100	29.81%	4428	48.64%
Somewhat weak	2571 (27.23%)	128	37.88%	2443	26.84%
Very weak	884 (9.37%)	84	25.06%	800	8.79%
Physical and functional health-related factors					
Chronic physical or mental health conditions (n = 9696)					
Yes	6336 (65.35%)	296	83.44%	6040	64.66%
No	3360 (34.65%)	59	16.56%	3301	35.34%
Functional impairment (n = 9267) Mean (SD)	6.60 (0.13)	15.70 (0.95)		6.28 (0.13)	

**Table 2:** Univariate survey-weighted logistic regression results: associations between COVID-19-related stressors, sociodemographic, mental and substance use disorders, psychosocial, and physical health factors, and suicidal ideation.

Variables	OR (95%CI)	*p*-Value
COVID-19 pandemic stressors		
Loneliness or emotional distress (ref: no)		
Yes	3.73 (3.32–4.19)	<0.001
Financial strain (ref: no)		
Yes	2.16 (1.97–2.37)	<0.001
Social demographic and economic factors		
Gender (ref: women+)		
Men+	0.80 (0.73–0.88)	<0.001
LGBTQ2+ status (ref: non-LGBTQ2+)		
LGBTQ2+	6.21 (5.56–6.93)	<0.001
Age (ref: ≥65 years)		
15 to 19 years	8.56 (7.11–10.30)	<0.001
20 to 24 years	7.72 (6.48–9.19)	<0.001
25 to 29 years	5.41 (4.36–6.70)	<0.001
30 to 34 years	4.83 (3.92–5.95)	<0.001
35 to 44 years	2.78 (2.27–3.40)	<0.001
45 to 54 years	2.56 (2.07–3.16)	<0.001
55 to 64 years	2.13 (1.73–2.63)	<0.001
Marital status (ref: married or living common law)		
Single	3.47 (3.13–3.84)	<0.001
Divorced or widowed	1.94 (1.63–2.31)	<0.001
Rurality (ref: less than 1000)		
Small population centre (1000 to 29,999)	0.99 (0.82–1.20)	0.948
Medium population centre (30,000 to 99,999)	1.56 (1.30–1.86)	<0.001
Large urban population centre (≥100,000)	1.08 (0.94–1.24)	0.271
Education (ref: less than high school)		
Highschool diploma/certificate	0.69 (0.60–0.79)	<0.001
Trade/college/university diploma/certificate	0.36 (0.31–0.43)	<0.001
Bachelor’s degree or higher	0.38 (0.33–0.44)	<0.001
Household income (ref: low income, i.e., <CAD 30,000)		
Middle income (CAD 30,000 to CAD 99,999)	0.90 (0.75–1.08)	0.269
High income (≥CAD 100,000)	0.76 (0.66–0.91)	0.002
Insurance coverage for prescription medicine (ref: no)		
Yes	0.87 (0.78–0.96)	0.004
Mental health and substance use disorders		
Mood disorders (ref: no)		
Yes	15.04 (13.68–16.55)	<0.001
Anxiety disorders (ref: no)		
Yes	8.45 (7.69- 9.28)	<0.001
Substance use disorders (ref: no)		
Yes	5.28 (4.60–6.05)	<0.001
Psychosocial factors		
Social belongings (ref: very strong)		
Somewhat strong	1.33 (1.13–1.57)	0.001
Somewhat weak	3.06 (2.60–3.61)	<0.001
Very weak	6.19 (5.18–7.40)	<0.001
Physical and functional health-related factors		
Chronic physical or mental health conditions (ref: no)		
Yes	2.75 (2.45–3.10)	<0.001
Functional impairment	1.06 (1.06–1.06)	<0.001

**Table 3:** Multivariable survey-weighted logistic regression results: associations between COVID-19-related stressors, sociodemographic, mental and substance use disorders, psychosocial, and physical health factors, and suicidal ideation.

Variables	aOR (95%CI)	*p*-Value
COVID-19 pandemic stressors		
Loneliness or emotional distress (ref: neither stressor)		
Yes	1.54 (1.29–1.84)	<0.001
Financial strain (ref: neither stressor)		
Yes	0.58 (0.43–0.80)	0.001
Both loneliness or emotional distress and financial strain (ref: neither stressor)		
Yes	2.05 (1.71–2.45)	<0.001
Social demographic and economic factors		
Gender (ref: women+)		
Men+	1.04 (0.93–1.17)	0.470
LGBTQ2+ status (ref: non-LGBTQ2+)		
LGBTQ2+	1.80 (1.54–2.12)	0.001
Age (ref: ≥65 years)		
15 to 19 years	3.85 (2.82–5.26)	<0.001
20 to 24 years	3.92 (2.95–5.20)	<0.001
25 to 29 years	2.52 (1.89–3.35)	<0.001
30 to 34 years	3.33 (2.54–4.37)	<0.001
35 to 44 years	1.79 (1.38–2.33)	<0.001
45 to 54 years	1.82 (1.39–2.39)	<0.001
55 to 64 years	0.86 (0.65–1.15)	0.310
Marital status (ref: married or living common law)		
Single	1.29 (1.07–1.55)	0.007
Divorced or widowed	1.85 (1.49–2.29)	<0.001
Rurality (ref: less than 1000)		
Small population centre (1000 to 29,999)	1.02 (0.81–1.30)	0.843
Medium population centre (30,000 to 99,999)	1.15 (0.91–1.46)	0.249
Large urban population centre (≥100,000)	0.82 (0.68–0.99)	0.040
Education (ref: less than high school)		
Highschool diploma/certificate	0.89 (0.72–1.09)	0.245
Trade/college/university diploma/certificate	0.66 (0.52–0.83)	<0.001
Bachelor’s degree or higher	0.91 (0.72–1.14)	0.401
Household income (ref: low income, i.e., <CAD 30,000)		
Middle income (CAD 30,000 to CAD 99,999)	0.82 (0.64–1.05)	0.120
High income (≥CAD 100,000)	0.87 (0.68–1.11)	0.268
Insurance coverage for prescription medicine (ref: no)		
Yes	0.83 (0.73–0.94)	0.004
Mental health and substance use conditions		
Mood disorders (ref: no)		
Yes	4.41 (3.79–5.13)	<0.001
Anxiety disorders (ref: no)		
Yes	1.83 (1.57–2.13)	<0.001
Substance use disorders (ref: no)		
Yes	1.50 (1.26–1.79)	<0.001
Psychosocial factors		
Social belongings (ref: very strong)		
Somewhat strong	1.51 (1.20–1.91)	0.001
Somewhat weak	1.62 (1.27–2.07)	<0.001
Very weak	2.81 (2.18–3.63)	<0.001
Physical and functional health-related factors		
Chronic physical or mental health conditions (ref: no)		
Yes	2.08 (1.79–2.43)	<0.001
Functional impairment	1.02 (1.01–1.03)	<0.001

**Table 5:** Pairwise comparisons of predicted probability of suicidal ideation. Note: Each comparison is written as (Financial Stress, Loneliness/Emotional Distress). “Yes” indicates the presence of COVID-related financial stress or loneliness/emotional distress; “No” indicates its absence. The table presents pairwise risk differences in predicted probability of suicidal ideation, adjusted for covariates.

Comparison (Financial Stress, Loneliness/Emotional Distress)	Contrast (Risk Difference)	Std. Error	*p*-Value	95% CI
(No, Yes) vs. (No, No)	0.0106	0.0022	<0.001	(0.0049, 0.0163)
(Yes, No) vs. (No, No)	−0.0090	0.0024	0.001	(−0.0153, −0.0027)
(Yes, Yes) vs. (No, No)	0.0195	0.0026	<0.001	(0.0128, 0.0263)
(Yes, No) vs. (No, Yes)	−0.0196	0.0026	<0.001	(−0.0266, −0.0127)
(Yes, Yes) vs. (No, Yes)	0.0089	0.0024	0.001	(0.0025, 0.0153)
(Yes, Yes) vs. (Yes, No)	0.0286	0.0029	<0.001	(0.0210, 0.0361)

## Data Availability

The data used in this study are publicly available and can be accessed through the 2022 Mental Health and Access to Care Survey.
